# Advancing the Chemical Characterization of *Eperua oleifera* Duke Oleoresin: A UHPLC-HRMS-Based Approach

**DOI:** 10.3390/plants14182893

**Published:** 2025-09-18

**Authors:** Rayssa Ribeiro, Gabriel Reis Alves Carneiro, Gustavo Ramalho Cardoso dos Santos, Márcio Vinícius da Silva Gomes, Henrique Marcelo Gualberto Pereira, Monica Costa Padilha, Valdir F. Veiga-Junior

**Affiliations:** 1 Military Institute of Engineering, Praça General Tibúrcio, 80, Praia Vermelha, Urca, Rio de Janeiro 22290-270, RJ, Brazil; rayssaribeiro_92@hotmail.com; 2 Brazilian Doping Control Laboratory (LBCD), Chemistry Institute (IQ), Federal University of Rio de Janeiro (UFRJ), Rio de Janeiro 21941-909, RJ, Brazil; gabriel.carneiro@iq.ufrj.br (G.R.A.C.); gustavo.santos@iq.ufrj.br (G.R.C.d.S.); marcio.gomes@iq.ufrj.br (M.V.d.S.G.); henriquemarcelo@iq.ufrj.br (H.M.G.P.); monicapadilha@iq.ufrj.br (M.C.P.)

**Keywords:** *Eperua oleifera* Ducke, UHPLC-HRMS, target and untargeted approach, diterpene acids, methyl esters of acid diterpenes

## Abstract

*Eperua oleifera* Ducke (Fabaceae), commonly known as *copaíba-jacaré*, is traditionally used for therapeutic purposes, like *Copaifera* oleoresins. Previous GC-MS studies reported its chemical composition as mainly composed of diterpenic acids, consistent with species of the same genus. Although GC-MS remains widely used for comparing compound retention times and fragmentation patterns, its application to diterpenic acids requires a derivatization step to form methyl esters due to the poor chromatographic performance of carboxylic acids on methyl silicone stationary phases. This step may lead to misinterpretations, especially considering recent findings of naturally occurring methyl esters in oleoresins that may co-elute with derivatized acids. This study aimed to apply more sensitive analytical techniques to identify both target and untargeted compounds. The resin of *E. oleifera* was analyzed by GC-MS to assess the presence of volatile components. Additionally, UHPLC-HRMS was employed using full-scan MS, data-dependent acquisition (DDA), and parallel reaction monitoring (PRM) in both positive and negative ESI modes. GC-MS confirmed the absence of volatile sesquiterpenes, classifying *E. oleifera* as a resin. Targeted UHPLC-HRMS detected natural methyl esters of diterpenic acids, while untargeted analysis using Compound Discoverer 3.3 software revealed flavonoids and phenolic compounds not previously reported. These findings support the application of UHPLC-HRMS as a powerful tool in phytochemical studies.

## 1. Introduction

*Eperua oleifera* Ducke (Fabaceae) is commonly known as “*copaíba-jacaré*” and is distributed in the Central Amazon, from Ecuador and Brazil to Guyana, and Venezuela [1,2]. Trees of the *Eperua* genus are known to share properties similar to those of another Fabaceae-Caesalpinoideae genus: *Copaifera*. Both exude a viscous oleoresin from the trunks of the trees, which is used for therapeutic purposes as a healing, antifungal, and antibacterial material [2]. However, despite the similarity in the therapeutic and morphological properties of these oleoresins, there are few reports focused specifically on *Eperua* species. Nevertheless, among the studied species, several classes of compounds were identified, including phenolic acids, flavonoids, sesquiterpenes, triterpenes, and, notably, diterpenes, which appear to be the most abundant [3,4].

Previously, our studies on the oleoresin of *Eperua oleifera* using Gas Chromatography coupled with Mass Spectrometry (GC-MS), as a standard analytical tool for analyzing terpene mixtures, after derivatization, led to the identification of three diterpene alcohols and nine diterpene acids (unpublished results). Typically, GC-MS analysis is performed following a derivatization reaction to produce the corresponding esters, due to the poor resolution of carboxylic acids on methyl silicone stationary phases. This introduces an additional analytical step that may introduce systematic errors and lead to the misinterpretation of results. As a result, carboxylic acids are commonly identified as their methyl esters, although naturally occurring methyl esters had not previously been reported in this type of oleoresin. Additionally, at the same previous study, phytochemical isolation using silica open column chromatography and traditional tools to natural products identification, such as multiple experiments using nuclear magnetic resonance (NMR), infrared and ultraviolet spectroscopy and direct insertion on high resolution mass spectrometry resulted on the unexpected description of methyl hardwickiate, a natural methyl ester not previously described in Amazon oleoresins (unpublished results). This stimulates further studies aimed at expanding the chemical knowledge of this oleoresin. Several questions arise from these findings, including the apparent absence of sesquiterpenoids or even monoterpenoids, which comprise the volatile and oily fraction of oleoresins; the potential presence of other chemical classes beyond traditional terpenoids; and the need to evaluate whether “auto”-esterification may occur during chromatographic processes, as well as to confirm the presence of additional diterpene esters alongside their corresponding diterpene carboxylic acids.

The usual method for identifying and analyzing specialized substances from more polar chemical classes, such as flavonoids, phenolic acids, and alkaloids, involves High-Performance Liquid Chromatography (HPLC) with ultraviolet or Diode Array Detection (HPLC-DAD), or Gas Chromatography paired with a Flame Ionization Detector (GC-FID) or GC-MS [5,6,7]. Studies employing HPLC-DAD have been used to quantify cinnamic acid derivatives and kaurene-type diterpenes in *Mikania laevigata* and *Mikania glomerata* [8]. Furthermore, a method developed using HPLC-DAD enabled the quantification of (-)-copalic acid in the oleoresin of *Copaifera langsdorffii* [5]. In all these methods, for the identification of diterpenic acids, an acidic modifier, such as phosphoric acid at concentrations ranging from 0.1% to 1.0% (*v*/*v*), was added to the eluents to facilitate the analysis of acidic diterpenes. Additionally, depending on the chemical structure of these compounds, HPLC-DAD or HPLC-UV techniques present detection limitations, as they may lack suitable chromophores.

The use of ultra-high-performance liquid chromatography coupled with high-resolution mass spectrometry (UHPLC-HRMS) analysis allows an entirely different perspective. While traditional methods can be slower and less sensitive, UHPLC-HRMS combines the fast and efficient separation of UHPLC with the high sensitivity and specificity of mass spectrometry, enabling faster, more accurate, and reliable analysis, especially in complex samples. Advances in liquid chromatography coupled with high-resolution mass spectrometry (LC-HRMS) have enabled both targeted and untargeted analyses across a wide range of complex matrices [9,10,11]. Several types of mass spectrometers, such as Time-Of-Flight (TOF), ion trap TOF, hybrid quadrupole TOF, and Orbitrap systems, routinely deliver high mass accuracy [10,12,13], allowing the determination of molecular formulas based on exact mass measurements. However, despite these technological advancements, compound identification remains a challenge. This is primarily due to the absence of many metabolites in reference databases, the wide dynamic range of metabolite concentrations, and limitations in the acquisition speed of mass spectrometry data. As a result, a significant number of detected peaks remain unidentified [10].

Software tools are available for compound detection, offered either as online platforms or as packages developed in programming languages such as R and Python [14,15,16,17]. Additionally, each mass spectrometer manufacturer provides its data processing software. The analysis of plant matrices produces complex results due to the vast diversity of naturally occurring compounds. In this context, Compound Discoverer has been utilized in untargeted metabolomics studies for the identification of compounds. This software is compatible with files generated by Orbitrap mass spectrometers and enables automated compound annotation through integration with the mzCloud database [18,19].

In this study, UHPLC-HRMS was employed using multiple data acquisition modes, including full mass spectrometry (Full MS), data-dependent acquisition (DDA), and parallel reaction monitoring (PRM), in both positive and negative ionization modes. Both targeted and untargeted analyses were applied as complementary approaches to advance the chemical characterization of the oleoresin from *Eperua oleifera* Ducke. The targeted analysis focused on detecting previously identified compounds from *Eperua* and *Copaifera*, comparing experimental data with reference parameters such as exact *m*/*z* values, retention times, and characteristic fragmentation patterns. In addition, the presence of sesquiterpenes was investigated over a broad range of detection limits using GC-MS. Meanwhile, the untargeted approach employed a metabolomics workflow to explore the potential of automated compound annotation using the Compound Discoverer 3.3 software exclusively. Finally, experiments were also conducted to evaluate the possibility of Fischer esterification occurring within the chromatographic system and whether this could lead to the formation of esters from diterpenic acids.

## 2. Results and Discussion

Oleoresins are characterized as complex mixtures composed of terpenoids from various classes. They typically consist of volatile liquid terpenoids, such as monoterpenes and sesquiterpenes, which act as solvents for heavier resinous terpenoids, including diterpenes and triterpenes, giving the material its characteristic viscous oil appearance [20]. Terpenes exhibit well-defined chemical and chromatographic characteristics as described in the literature. In GC-MS analyses, for example, they elute within specific temperature ranges depending on their class; sesquiterpenes typically elute between 120 °C and 200 °C [21]. These elution patterns are closely related to the molecular weights of the compounds and their corresponding temperature intervals. Such correlations enable a preliminary analysis of oleoresins by linking terpene classes to their characteristic elution temperatures. The first question addressed became evident. In the absence of monoterpenes and sesquiterpenes, the volatile liquid fraction of *E. oleifera* should no longer be considered to produce oleoresins (or oilresin) and would be instead classified only as a resin.

A previous study on *Eperua oleifera* oleoresin, using GC-MS after derivatization, detected diterpene acids commonly found in both *Copaifera* and *Eperua* oleoresins, and also isolated a natural methyl ester. A derivatization step is commonly employed for the GC-MS analysis of oleoresins, especially when using 5%-phenyl-methylpolysiloxane columns, since the chromatographic resolution of acidic compounds is often inadequate without derivatization. However, this procedure can lead to compound misidentification, as it does not allow for the differentiation between naturally occurring methyl esters and methyl esters formed from derivatized diterpene acids. These findings, combined with the recent isolation of methyl hardwickiate (unpublished results) provide further support for a targeted search for other diterpene acids and their corresponding methyl esters using more sensitive analytical techniques, such as UHPLC-HRMS. This approach enables the detection of compounds in high mass resolution and present at low concentrations that may not be detectable by GC-MS. Furthermore, it allows us to address a key question: whether the oleoresin (or resin) from *E. oleifera* naturally produces a diversity of diterpene methyl esters alongside diterpene acids, as previously suggested in the literature.

**Figure 1:**
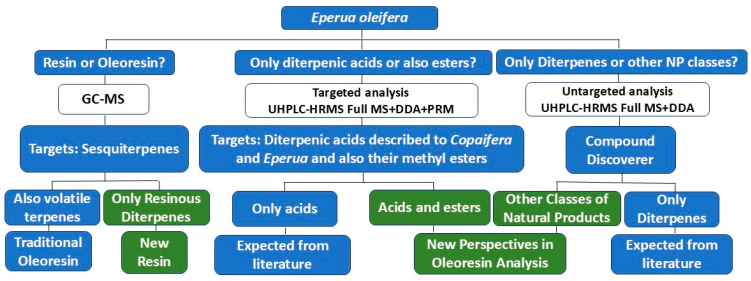
Analytical strategy applied to the study of *Eperua oleifera*.

The third question of this study is to expand the chemical knowledge on *E. oleifera*. Since natural diterpenoid esters were never detected before in this oleoresin, and terpenes are typically characterized as its main constituents, should other substances, from different natural biosynthetic classes, be present? The oleoresin of *E. oleifera* exhibits the physical characteristics of an oil, raising questions about the possible contribution of other compounds to its oily appearance. Analytical tools and software platforms such as Compound Discoverer are instrumental in the search for previously unidentified targets (Figure 1).

### 2.1. Evaluation of Sesquiterpene Presence Using GC-MS

**Figure 2:**
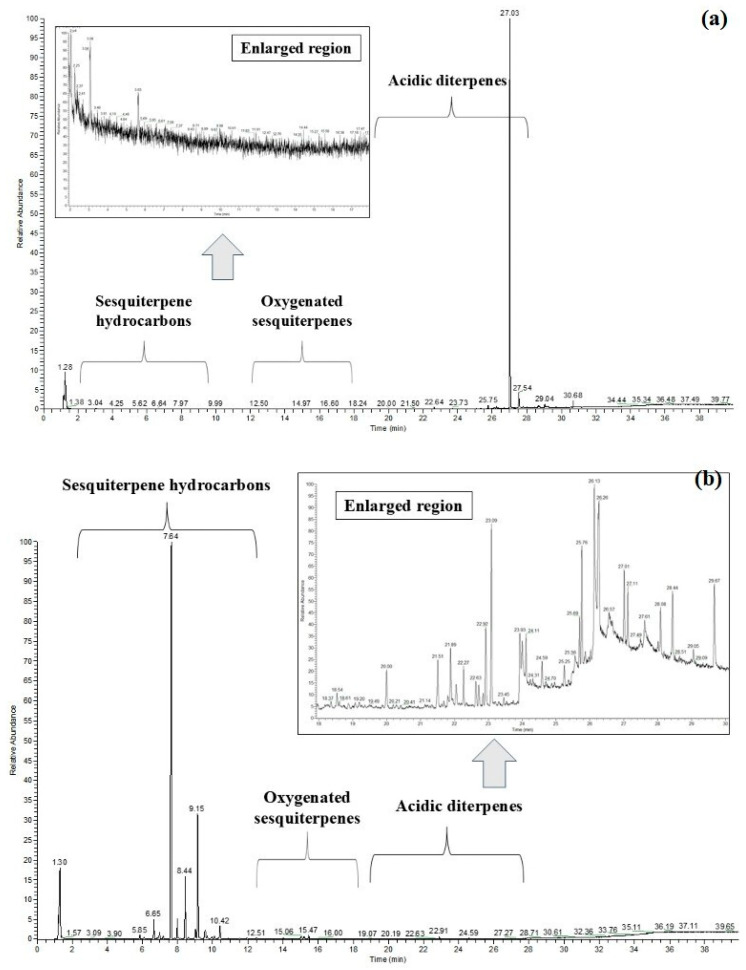
Chromatograms of *Copaifera multijuga* (**a**) and *Eperua oleifera* (**b**) oleoresins.

To verify the presence of sesquiterpenes in the oleoresin of *E. oleifera*, the oleoresin of *C. multijuga* was used as a reference due to the extensive amount of information available regarding the chemical characterization of this species and the amount of sesquiterpene present, relating to the diterpenic acids [21,22]. Using GC-MS, it was possible to obtain a structured chromatogram based on the chemical profile of the oleoresins. The structured chromatogram is divided into three distinct regions: the sesquiterpene hydrocarbon region, the oxygenated sesquiterpene region, and the acidic diterpene region. Figure 2a illustrates the total ion chromatogram of the oleoresin from *C. multijuga*.

The first region of the chromatogram (Figure 2) corresponds to sesquiterpene hydrocarbons, which elute between 6 and 11 min. The main compounds identified in this region include α-copaene (6.62 min), β-caryophyllene (7.64 min), β-humulene (8.43 min), and β-bisabolene (9.96 min). The second region consists of oxygenated sesquiterpenes, eluting between 12 and 18 min. Notably, caryophyllene oxide elutes at 12.48 min within this region. In the final region, acidic diterpenes elute between 19 and 29 min. Among them, copalic acid (25.76 min), a labdane-type diterpenic acid, is considered a biomarker for species of the *Copaifera* genus.

The same analytical approach was applied to the *E. oleifera* oleoresin. No sesquiterpenes, neither hydrocarbon nor oxygenated forms, were detected; only acidic diterpenes and their corresponding esters were identified. Figure 2b shows the total ion chromatogram (TIC) of *E. oleifera* oleoresin. An expanded view of the sesquiterpene elution region is also provided to confirm that no sesquiterpenes were detected within the method’s detection limits. The absence of volatile compounds such as sesquiterpenes in *E. oleifera* supports the hypothesis that this material should be classified as a resin rather than an oleoresin, suggesting the need to investigate other compounds that may account for its semi-liquid or viscous appearance, rather than a purely solid form.

### 2.2. Chemical Characterization of Diterpenes (Targeted) by UHPLC-HRMS

The chemical composition of *E. oleifera* was investigated using UHPLC-HRMS in both negative and positive electrospray ionization (ESI) modes. Through a targeted analysis approach, eleven diterpenic acids and six of their corresponding methyl esters were identified (Table 1). The UHPLC-HRMS experiment was conducted using Full MS, DDA, and PRM acquisition modes to identify the target compounds: diterpenic acids and their corresponding methyl esters (hardwickiate, patagonate, copalate, agathate, acetoxycopalate, and eperuate). The analytes were identified based on their exact masses (mass error < 0.5 ppm), and their elution order was suggested. According to Sumner et al. (2007), compounds that are considered to belong to a specific chemical class, based on characteristic physicochemical properties or spectral similarity to known members of that class, are assigned to confidence level 3 at the metabolomic approach to compound identification [23]. During the PRM acquisition, the analytes identified in the targeted approach were fragmented using HCD (collision energy of 40 eV). With this collision energy, it was possible to obtain the main fragments for the diterpene acids and their respective methyl esters, as described in the present study (Table 1).

To ensure effective ionization of the acids, formic acid and ammonium formate were used as mobile phase modifiers. This combination enhances the ionization of weak acids and bases, enabling ESI analysis while significantly improving peak resolution and separation. The mobile phase pH had a notable impact on retention times and chromatographic peak shapes, as it influenced the ionization state of the analytes. For diterpenic acids, most are better separated under slightly basic conditions where the acidic analytes are ionized. Solvent composition, acidity, and analyte polarity are key factors influencing ionization efficiency in negative ESI MS mode [10,24].

For fragmentation, the analytes are infused into the ESI source, which promotes protonation or deprotonation and/or the formation of adducts. The even-electron ions (EE^+^ or EE^−^) typically display low internal energy content; consequently, few or no fragments are observed in the mass spectra of these ions.

In ESI, particularly for organic molecules, typical losses observed during fragmentation include neutral molecule losses (H_2_O, −18 Da; CO, −28 Da; CO_2_, −44 Da; CH_3_OH, −32 Da; C_2_H_4_O, −44 Da); cleavages such as Retro-Diels–Alder (RDA), cleavage of glycosidic bonds, and formation of proton adducts (M+H in positive ESI mode and M−H in negative mode), as well as sodium, potassium, and ammonium adducts ([M+Na]^+^, [M+K]^+^, and [M+NH_4_]^+^).

About fragmentation of diterpenic acids in negative ion mode (ESI^−^), as hardwickiic acid can be observed, neutral losses characteristic of carboxylic acids, notably CO_2_ (−44 Da), forming the [M−H−CO_2_]^−^ species. Additional losses of H_2_O (−18 Da) and CO (−28 Da), can arise due to hydroxylated or methoxylated sites common in the diterpene structure. Table 1 presents specific fragments for hardwickiic acid, other diterpenic acids, and methyl ester correspondents that are not yet described in the literature. Well established expectations are for a neutral loss of CO_2_ (–44 Da) as the dominant fragment, potentially accompanied by losses of H_2_O, CO, or CH_3_OH, depending on the functional groups present. These neutral losses and potential ring cleavage pathways are consistent with documented fragmentation behavior of similar carboxylic terpenoids under negative ESI-CID or HCD conditions. However, no fragmentation data for hardwickiic acid in ESI-negative mode have been reported in the literature. The only available data pertains to GC-EI spectra in SpectraBase. These fragmentation pathways are consistent with analogous diterpenoids and can support the tentative annotation of the compound under study.

The limited studies available on *Eperua* species indicate that labdane-type diterpenic acids are the most frequently identified [3,25]. As the resins of *Eperua* species are often described as being similar to those of the *Copaifera* genus, our findings further support this connection, given that most of the compounds identified in this study have been reported in both genera [26,27,28]. The main acids found include hardwickiic, dihydroagathic (pinifolic), agathic, and copalic—all of which are also found in copaiba oils. Among the methyl esters, methyl hardwickiate was the most abundant. The ratio between hardwickiic acid and its methyl ester was approximately 1.3.

Oleoresins are traditionally used for medicinal purposes in northern Brazil, with knowledge passed down through native populations. However, such uses still lack scientific validation. Chemical characterization helps bridge traditional knowledge with scientific understanding. Notably, some of the identified diterpenes, such as hardwickiic acid and eperuic acid, have demonstrated antitumor, anti-leishmania, and anti-inflammatory activities [29,30,31,32,33]. Both compounds are present in *E. oleifera*.

Although the evidence is compelling—given that the analysis involves acids in a mobile phase containing methanol (an alcohol) and acidic additives, which could promote ester formation through the well-known Fischer esterification reaction additional experiments were conducted to assess the influence of the medium (mobile phase) on the analysis of diterpenic acids in oleoresins solubilized in methanol.

#### 2.2.1. Evaluation of the Kinetics of Diterpenoate Methyl Ester Formation

The Fischer esterification is a method for forming esters from carboxylic acids and alcohols in the presence of an acid catalyst. The equilibrium is driven toward the ester product by using a substantial excess of alcohol. To evaluate the likelihood of this reaction occurring in our system, control experiments were conducted under the same chromatographic conditions but with variations in solvent composition. The oleoresin analysis repeated using a mobile phase devoid of the additives formic acid and ammonium formate made it impossible to detect the diterpene acids. In ESI, the first step to ensure detection is ionization; once the analytes are ionized, volatilization occurs, followed by detection. This experiment demonstrates the necessity of additives for the effective detection of diterpene acids.

##### Oleoresin Dissolved in Methanol Containing 0.1% Formic Acid

Based on the results described in Section 2.1 and considering the characteristics of electrospray ionization, a new experiment was conducted to evaluate the possible occurrence of Fischer esterification within the vial. To this end, following the sample preparation procedure outlined in Section 3.3.1, we assessed the potential formation of esters by analyzing the oleoresin sample, which was solubilized in methanol containing 0.1% formic acid, at pre-established time intervals (Table 2).

The data presented in Table 2 demonstrate that the medium does not catalyze the formation of any methyl ester. The area values obtained for both the diterpene acids and their corresponding esters show a coefficient of variation of less than 10%, indicating the repeatability of the measurement. If ester formation were occurring, we would expect to observe a progressive decrease in the acid peak areas, accompanied by an increase in the ester peak areas.

##### Oleoresin Dissolved in Acetonitrile

To evaluate whether the methyl group of the ester could be coming from methanol, we repeated the assessment of the kinetics of methyl ester formation from acidic diterpenes using acetonitrile as the solvent. Table 3 supports the findings from the experiment described in item 3.2.1, confirming that the medium does not promote the formation of methyl esters.

From Table 3, it is possible to observe a reduction of approximately one order of magnitude in all area values obtained for the samples dissolved in acetonitrile compared to those dissolved in methanol. The greater tendency for ionization easily explains this result, and thus detection by ESI, when methanol with 0.1% formic acid is used as the dilution solvent.

### 2.3. Putative Identification of Other Substances in E. oleifera Resin Using the UHPLC-HRMS Approach

High-resolution mass spectrometry offers greater mass accuracy, enabling the identification of a broader range of compounds compared to other techniques. Both positive and negative ion modes were recorded using UHPLC-Q-Orbitrap HRMS. The untargeted approach, performed with Compound Discoverer 3.3 software, enabled the detection of compounds by comparing fragmented data with known fragmentation rules. The workflow used for compound annotation is described in Section 3.3.2.

Alongside some non-targeted diterpenes (Table 1), other classes of natural products not previously reported in oleoresins were tentatively identified, including flavonoids, benzoquinones, triterpenes, and phenolics, among others (Table 4). It is important to emphasize that Compound Discoverer infers substances by combining chromatographic data with fragmentation patterns (pseudomolecular ion and MS^2^ data). For isomeric compounds, multiple peaks were observed, and their fragmentation patterns were compared with those in the mzCloud and ChemSpider databases, as described in Section 3.3.2. Table 4 shows the compound assignments in both ESI positive and negative modes, including mass errors and the molecular formulas identified in the oleoresin.

Among the classes of natural products detected, flavonoids and phenolic acids have been reported as chemical constituents in the heartwood of *Eperua falcata* [34]. Additionally, triterpenes have been identified in the leaves of *Eperua bijuga* [35].

The untargeted study utilizing tools that enable compound-focused searches led to the detection of compounds not typically reported in oleoresins or resins. For isomers, the software, combining spectral library data with information about the analytical conditions, infers the substance’s identity. For example, luteolin and kaempferol, respectively, a flavone and a flavonol, are constitutional isomers, sharing the same molecular mass and, therefore, the same pseudomolecular ion. In electrospray ionization mass spectrometry (ESI-MS), the fragment ions (product ions) produced from the pseudomolecular ion are similar but show different relative intensities. In kaempferol, the hydroxyl group at C-3 (ring C) is replaced by a hydrogen atom in luteolin [36]. These structural differences influence the stability and fragmentation pathways, changing the abundance or relative intensities of the fragments. The structural variation also slightly impacts the polarity of the analytes and, consequently, their retention time. For luteolin, the *m*/*z* 151 and *m*/*z* 133 fragments are generated, with *m*/*z* 151 being the most intense [37]. This cleavage occurs through a Retro Diels Alder (RDA) reaction. For kaempferol, the *m*/*z* 153 fragment is the most intense, and the hydroxyl group at C-3 favors its formation [38].

Although luteolin and kaempferol share the same exact mass and produce similar fragment ions, their MS/MS relative abundance patterns differ depending on collision energy and chromatographic conditions. In our work, we combined retention time, collision energies, and database/library matching to annotate these flavonoids; nevertheless, without authentic standards, these annotations remain putative (MSI level 2).

In the case of (+)-catechin and (−)-epicatechin, they are stereoisomers (epimers) that share the same mass ([M–H]^−^ ≈ *m*/*z* 289) and produce largely overlapping MS/MS fragmentation patterns. Common product ions reported in the literature for both compounds include *m*/*z* 245 (loss of 44 Da), *m*/*z* 203, *m*/*z* 179, *m*/*z* 151, *m*/*z* 137, and *m*/*z* 125. Because these ions are shared, differentiation by MS/MS is based mainly on subtle differences in relative abundances across collision energies and chromatographic behavior rather than on unique diagnostic fragments. 

The difference in the configuration at C-3 of the C-ring affects the three-dimensional conformation of the molecule, influencing hydrogen bonding and the stability of the resulting product ions during fragmentation. As a result, both compounds generate similar MS/MS fragments, but the relative intensities of diagnostic ions differ. This stereochemical variation can also slightly alter the polarity of the analytes, resulting in minor differences in retention times during chromatographic separation.

This approach, using more sensitive analytical techniques, provided new insights into the chemical profile of resins that had previously gone unrecognized due to the limitations of earlier methodologies.

## 3. Materials and Methods

### 3.1. Plant Material

The oleoresin from *Eperua oleifera* Ducke was collected on 6 June 2023, in Manicoré, Amazonas, Brazil. The access was registered under code A9F18E3 in the SISGEN system. The oleoresin from *Copaiba multijuga* Ducke was collected in Manaus, Amazonas, Brazil. The access was registered under code AAB3AA1 in the SISGEN system. The sample preparation for UHPLC-HRMS was performed with approximately 1 mg of each oleoresin weighed into a vial and solubilized with 1 mL of methanol.

### 3.2. GC-MS Analysis and Instrument Conditions

A Thermo Scientific 1300 Trace Gas Chromatograph coupled with an ISQ LT single quadrupole MS in a DB-5HT column of 30 m × 0.250 mm ID and 0.10 µm film thickness, 5%-phenyl-methylpolysiloxane. Pulsed Split injection mode was selected to inject 3 µL of sample into the inlet liner Ultra Inert, splitless, single taper, glass wool (Agilent Part number: 5190-3171, 4 mm × 900 µL) at 270 °C, using helium as the carrier gas at flow rate of 1.0 mL/min and split ratio of 20:1. The injection pulse pressure was set to 50.0 psi (3.960 mL/min) for 0.30 min, rate 1 mL/min, whereas the purge flow was set to 0.800 mL/min (injection mode: pulsed split). The initial temperature ramp of the oven started at 110.0 °C for 2 min and increased to 130.0 °C (for 5 min) at a rate of 3.0 °C/min, followed by a rise to 310.0 °C at 8.5 °C/min, and then held for 5 min. The total running time was 39.84 min. The MS transfer line temperature was set at 320 °C, and the ion source temperature was kept at 300 °C. The system was operated in EI mode at an energy level of 70 eV. The chromatogram was scanned in SCAN mode, with a mass range of *m*/*z* 50 to *m*/*z* 700. The mass spectra were interpreted using the reference library of the National Institute of Standards and Technology (NIST, Gaithersburg, MD, USA), along with literature data on previously identified diterpenes [39].

### 3.3. UHPLC-HRMS Analysis and Instrument Conditions

A Dionex Ultimate 3000 ultra-high performance liquid chromatography (UHPLC) system coupled to a QExactive Plus hybrid quadrupole Orbitrap mass spectrometer (Thermo Fisher Scientific, Bremen, Germany) equipped with an electrospray ionization (ESI) source was used. Separation was performed in a reversed-phase column (kinetex 2.6 µm PS C18, 100 Å, 100 mm × 2.1 mm; 2.6 µm) at 40 °C, with a constant flow rate of 300 μL/min and injection volume of 5 µL. A gradient chromatographic run started at 5% of mobile phase B (methanol with 0.1% formic acid) and 95% of mobile phase A (water with 5 mM ammonium formate and 0.1% formic acid). Mobile phase B increased to 10% at 1.0 min, 25% at 2 min, and 90% at 10 min. After reaching 100% of B at 14 min and maintaining this ratio until 16 min, the initial chromatographic condition was restored from 16.1 to 20.0 min.

The LC effluent was pumped to the mass spectrometer operating in a negative ESI mode, calibrated daily with a manufacturer’s calibration solution (Thermo Fisher Scientific, Bremen, Germany). ESI parameters were further optimized with the final setup: spray voltage of 2.9 kV, S-lens voltage of 80 V, the capillary temperature of 380 °C, auxiliary gas heater temperature of 350 °C, nitrogen sheath, auxiliary, and sweep gas were set at 30, 10, and 1 arbitrary units, respectively. The strategy of acquisition was Full-scan and Data Dependent Analysis (DDA), at the same time, in a range of *m*/*z* 70–*m*/*z* 1050 at a resolution of 70,000 full widths at half maximum (FWHM), automatic gain control (AGC) of 1 x 10^6^, and maximum injection time (IT) of 100 ms.

For the target compound identification study, a full MS scan approach was employed. The exact mass-to-charge (*m*/*z*) values of the detected targets were as follows: *m*/*z* 315.1966 ([M–H]^−^) to hardwickiic acid (C_20_H_28_O_3_), *m*/*z* 331.1915 ([M–H]^−^) to patagonic acid (C_20_H_28_O_4_), *m*/*z* 303.2330 ([M–H]^−^) to copalic acid (C_20_H_32_O_2_), *m*/*z* 333.2071 ([M–H]^−^) to agathic acid (C_20_H_30_O_4_), *m*/*z* 319.2278 ([M–H]^−^) to β-hydroxy-copalic acid (C_21_H_34_O_3_), *m*/*z* 335.2227 ([M–H]^−^) to dihydroagathic (pinifolic) acid (C_20_H_32_O_4_), *m*/*z* 305.2486 ([M–H]^−^) to eperuic acid (C_20_H_34_O_2_), *m*/*z* 303.2329 ([M–H]^−^) to kolavenic acid (C_20_H_32_O_2_), *m*/*z* 335.2227 ([M–H]^−^) to clerod-3-3n-15,18-dioic acid (C_20_H_32_O_4_), *m*/*z* 293.1758 ([M–H]^−^) to 14,15,16-*trinor*-hardwikiic acid (C_17_H_26_O_4_), *m*/*z* 317.2122 ([M–H]^−^) to 2-oxokolavenic acid (C_20_H_30_O_3_), *m*/*z* 329.2122 ([M–H]^−^) to methyl ester of hardwickiic acid or methyl hardwickiate (C_21_H_30_O_3_), *m*/*z* 317.2486 ([M–H]^−^) to methyl ester of copalic acid or methyl copalate (C_21_H_34_O_2_), *m*/*z* 375.2541 ([M–H]^−^) to acetoxy copalic acid methyl ester (C_23_H_36_O_4_) *m*/*z* 345.2071 ([M–H]^−^) to methyl ester of patagonic acid or methyl patagonate (C_21_H_30_O_4_), *m*/*z* 371.2227 ([M–H]^−^) to mono methyl ester of agathic acid or methyl agathate (C_21_H_34_O_4_), and *m*/*z* 319.2642 ([M–H]^−^) to methyl ester of eperuic acid (C_21_H_36_O_2_).

The second set of experiments was conducted using the Full MS and DDA approach, with the same gradient chromatographic run, employing methanol as mobile phase B and water as mobile phase A, without the addition of any additives.

Targeted mass spectrometry-based approaches were performed using the parallel reaction monitoring technique (PRM), the precursor ions were fixed at a resolution of 17,500 full width at half maximum (FWHM), automatic gain control (AGC) of 1 × 10^6^, maximum injection time (IT) of 100 ms, and quadrupole isolation window of *m*/*z* 2. In the PRM approach, the precursor ions were fragmented in a higher energy collisional dissociation (HCD) cell with (N)CE of 40%, as described in Table 4.

Data were acquired and processed using Thermo Scientific TraceFinder 4.1 software (Thermo Fisher Scientific, Austin, TX, USA), with a mass tolerance of ±5 ppm.

#### 3.3.1. Evaluation of the Kinetics of Methyl Ester Formation by UHPLC-HRMS

To evaluate the kinetics of methyl ester formation of acidic diterpenes, in the first experiment, 1 mg of each resin was weighed and dissolved in methanol containing 0.1% formic acid. In the second trial, the sample was dissolved in acetonitrile. The samples were analyzed with approximately 20-day intervals between the first and fourth analyses, and the coefficient of variation was calculated.

#### 3.3.2. Data Processing and Data Analysis Workflow

The chemical composition was compiled using online databases (mzCloud and ChemSpider) and imported into the Compound Discoverer 3.3 analysis platform to identify chromatographic peaks. The binary sample model was employed for comparative analysis, such as that between blank solvent samples and Quality control samples. Additionally, [M+H]^+^, [M+Na]^+^, and [M+NH_4_]^+^ were selected as the primary protonated and adduct ion modes in positive ionization mode, while [M–H]^−^ and [M–H–H_2_O]^−^ were chosen as the primary deprotonated and adduct ion modes in negative ionization mode. The upper and lower limits of molecular weight deviation were set to 5 ppm.

The data analysis with annotation is designed to annotate chemical compounds through peak extraction by characteristic ions screening and library matching. The workflow data is divided into the following steps: data acquisition, information extraction, MS/MS compound matching, MS library matching, and data integration.

In the data acquisition step, a combination of full-MS scan and ddMS2 methods was used to collect data for the blank and test samples. During the information extraction process, the precursor ion list was created by processing the datasets mentioned earlier. The precursor ions table (*m*/*z*, Rt, CE) was generated, and the program extracted the optimal MS/MS information for each ion based on the CE value. All data produced was manually verified. MS/MS–compound matching summarized the diagnostic and characteristic ions according to the fragmentation pathways. The *m*/*z* deviation was set to 5 ppm. In the MS library, known compounds reported in the literature are classified as the natural compound library. Using these tables and the step results as input, ions were further matched with the compound library based on *m*/*z*, retention time, and compound type. The matching criteria required that the deviation in *m*/*z* be within 5 ppm, the retention time deviation be less than 0.3 min, and the compound types be consistent. In the data integration step, the compound type may be predicted by diagnostic ions and matched with natural substances. When prediction by diagnostic ions is not possible, matching is performed with natural compounds or compounds that match the theoretical database.

## 4. Conclusions

The issues that were clarified by the chromatographic and spectrometric analysis evidenced that the *Eperua oleifera* does not present volatile compounds such as sesquiterpenes, which characterize oleoresins. So, *E. oleifera* exudation should be classified only as a resin, as it contains only the non-volatile diterpenic acid fraction. The development and application of a method using Full MS, Data-Dependent Acquisition (DDA), and Parallel Reaction Monitoring (PRM) acquisition modes on UHPLC-HRMS enabled the direct and simultaneous detection of acidic diterpenes and their methyl esters in the resin. Resinoid esters are rarely described in oleoresins. For effective ionization, the mobile phase modifiers formic acid and ammonium formate enhanced both electrospray ionization (ESI) efficiency and chromatographic resolution. The UHPLC-HRMS method highlighted the critical role of additive concentration in optimizing ESI and method accuracy. Key diterpenic acids identified included hardwickiic, dihydroagathic, agathic, and copalic acids, all of which are also found in *Copaifera* oleoresins. Among the methyl esters, methyl hardwickiate was the most abundant. Experiments using alternative solvents for both sample preparation and mobile phases confirmed that the observed methyl esters are naturally present and not artifacts from esterification during analysis. Finally, untargeted studies using Compound Discoverer software revealed the presence of flavonoids and phenolic acids not previously reported in resins or oleoresins, offering new insights into the chemical complexity of *E. oleifera*.

## Figures and Tables

**Table 1:** Diterpenes detected by UHPLC-HRMS and their parameters.

Compound	Molecular Formula [M]	Retention Time (min)	[M–H]^−^/[M+H]^+^	(N)CE (%)	Fragment Ions/Intensity (%)
Hardwickiic acid	C_20_H_28_O_3_	12.95	315.1966	40	301.1820 (14%)/285.18582 (11%)/273.2223 (26%)/257.1909 (16%)
Patagonic acid	C_20_H_28_O_4_	11.29	331.1915	40	287.2019 (100%)/259.2066 (34%)/243.1752 (28%)
Copalic acid	C_20_H_32_O_2_	14.00	303.2330	40	285.1861 (5%)/259.2069 (10%)
Agathic acid	C_20_H_30_O_4_	11.95	333.2071	40	301.1809 (66%)/289.2177 (28%)
Dihydroagathic (pinifolic) acid	C_20_H_32_O_4_	12.22	335.2227	40	303.1963 (40%)/285.1845 (100%)/245.1908 (88%)
Eperuic acid	C_20_H_34_O_2_	14.06	305.2486	40	287.2377 (10%)
Kolavenic acid	C_20_H_32_O_2_	14.02	303.2329	40	285.1861 (5%)/243.1754 (5%)
Clerod-3-en-15,18-dioic acid	C_20_H_32_O_4_	11.49	335.2227	40	303.1963 (40%)/285.1858 (100%)/275.1908 (46%)
14,15,16-trinor-hardwikiic acid **	C_17_H_26_O_4_	10.90/14.43	293.1758	40	249.1853 (20%)
2-oxokolavenic acid	C_20_H_30_O_3_	11.84	317.2122	40	273.2226 (20%)/285.1858 (12%)
Methyl hardwickiate	C_21_H_30_O_3_	13.90	329.2122	40	301.1802 (2%)/285.1855 (20%)/257.1905 (100%)
Methyl copalate	C_21_H_34_O_2_	14.90	317.2486	40	285.1862 (48%)/257.1913 (10%)
Methyl 3β-hydroxy copalate	C_21_H_34_O_3_	12.81	333.2435	40	301.1807 (10%)/273.2227 (20%)/257.1912 (25%)
Methyl 3β-acetoxy copalate	C_23_H_36_O_4_	13.88	375.2541	40	317.2121 (12%)/301.1804 (5%)/287.1655 (25%)
Methyl patagonate	C_21_H_32_O_4_	13.89	345.2071	40	301.2173 (10%)/243.1753 (32%)/255.1749 (40%)
Methyl agathate	C_21_H_32_O_4_	12.29	347.2227	40	319.1899 (7%)/303.1966 (100%)/287.1635 (10%)/243.1754 (39%)
Methyl eperuate	C_21_H_36_O_2_	14.95	319.2642	40	301.2170 (5%)/289.2173 (10%)
Cativic acid *	C_20_H_34_O_2_	14.10	305.2486	40	261.18573 (2%)
8,17-dihydroxy-13-labden-16,15-olid-19-oate *	C_21_H_32_O_6_	12.21	439.2340 [M-H-60]^−^	30	357.2052 (15%)
Effusanin A *	C_20_H_28_O_5_	10.84	347.1865	30	319. 1899 (3%)/303.1969 (37%)/259.2072 (11%)
18-hydroxy-clerod-3-en-15-oic acid *	C_20_H_34_O_3_	13.13	321.2437	30	277.2171 (60%)/247.2069 (14%)
Craterellin A *	C_22_H_34_O_4_	13.16	380.2792 [M+NH_4_]^+^	30	336.2792 (32%)/296.2474 (18%)
14-deoxy-11,12-didehydroandrographolide *	C_20_H_28_O_4_	11.94	315.1953 [M+H]-18	30	297.1845 (45%)/283.1699 (32%)
12-hydroxy-7-carboxy- abiet-8(13)-en-18-oic acid *	C_20_H_30_O_4_	12.53	335.2216	30	317.2111 (60%)/303.1960 (90%)
Aphidicolin *	C_20_H_34_O_4_	12.08	339.2529	30	295.2529 (60%)/277.2530 (32%)
7-keto, 12-hydroxy, abiet-8-14-en-18-oic acid	C_20_H_30_O_4_	12.71	333.2071	30	301.1803 (10%)/289.2176 (8%)
(-)-7β-hydroxycleroda-8(17),13E-diene-15-oic acid *	C_20_H_32_O_3_	13.47	319.2278	30	275.2381 (60%)
16-oxo-13,14H-hardwikiic acid *	C_20_H_28_O_4_	11.26	331.1914	30	287.2016 (100%)/243.1748 (10%)
Nor-hardwikiic acid *	C_17_H_26_O_4_	12.16	293.1758	30	249.1853 (100%)
7-oxo-labda-8-ene-15-oic acid *	C_20_H_30_O_3_	11.86	317.2122	30	285.1861 (5%)/273.2218 (100%)
(-)-cleroda-7,13E-diene-15-oic acid *	C_20_H_32_O_2_	14.62	303.2329	30	285.18536 (74%)/259.16934 (39%)
6β,7β-dihydroxykaurenoic acid *	C_20_H_30_O_4_	11.48	333.2071	30	301.18033 (70%)/289.21765 (32%)
8-hydroxyoctadeca-9,12-dienoic acid *	C_18_H_32_O_3_	13.86	295.2278	30	251.23836 (5%)/155.14264 (5%)
Ent-16β,17-dihydroxy-19-kaurenoic acid *	C_20_H_32_O_4_	13.05	335.2227	30	291.23301 (40%)/273.2227 (32%)

* Untargeted approach, putative identification; ** isomers.

**Table 2:** Area of the diterpene acids and their respective methyl esters (in methanol).

Experiment Dates	Target Analytes or Target Substances															
	Hardwickiic Acid	CV%	Methyl Hardwickiate	CV%	Copalic Acid	CV%	Methyl Copalate	CV%	Patagonic Acid	CV%	Methyl Patagonate	CV%	Agathic Acid	CV%	Methyl Ester of Agathic Acid	CV%
15 May 2024	1,975,121,726	8.0	1,512,316	5.1	1,203,063,795	7.7	921,009	4.4	74,788,718	7.7	891,007	5.2	399,396,558	7.5	509,843	6.2
20 May 2024	1,926,711,350		1,341,619		1,114,681,593		870,510		64,791,889		862,986		406,791,632		498,032	
25 May 2024	1,711,628,436		1,470,515		122,3025,777		941,617		74,937,278		789,675		453,361,272		569,872	
4 June 2024	2,075,347,716		1,421,008		1,098,075,485		858,979		77,429,128		871,585		381,595,128		543,929	

**Table 3:** Area of the diterpene acids and their respective methyl esters (in acetonitrile).

Experiment Dates	Target Analytes or Target Substances															
	Hardwickiic Acid	CV%	Methyl Hardwickiate	CV%	Copalic Acid	CV%	Methyl Copalate	CV%	Patagonic Acid	CV%	Methyl Patagonate	CV%	Agathic Acid	CV%	Methyl Ester of Agathic Acid	CV%
15 May 2024	177,534,771	6.0	122,541	6.1	109,567,508	5.2	76,870	8.5	7,278,871	7.6	79,100	8.5	37,939,253	4.9	48,209	4.1
20 May 2024	169,568,903		130,981		152,698,547		64,191		6,479,188		75,298		39,891,163		47,981	
25 May 2024	162,671,135		131,701		191,469,162		67,078		7,093,297		75,465		40,459,027		50,629	
4 June 2024	186,713,428		142,216		100,330,879		65,132		7,798,235		89,698		36,289,712		52,298	

**Table 4:** Analytes detected using an untargeted approach of *E. oleifera* by UHPLC-HRMS.

Class of Natural Products	Substance Detected	Molecular Formula [M]	[M-H]^−^/ [M+H]^+^	Fragment Ions
Polyacetylene	(R)-(-)-Falcarinol	C_17_H_24_O	243.1753	181.15877 (10%)/155.08554 (8%)
Benzoquinone	5-O-ethyl embelin	C_19_H_30_O_4_	321.2073	277.2167 (100%)/247.2060 (15%)
	Embelin	C_17_H_26_O_4_	293.1758	249.1758 (100%)/219.1758 (10%)
Fatty Acid	Methyl palmitate	C_17_H_34_O_2_	288.2893	220.9344 (11%)/90.9771 (100%)
	(13Z)-8-hydroxyoctadecene-9,11-diynoic acid	C_18_H_26_O_3_	289.1812	256.9605 (10%)/156.9426 (5%)
	α-Linolenic acid	C_18_H_30_O_2_	277.2174	247.2061 (25%)/259.2041 (10%)/123.0077 (15%)
	Ricinoleic Acid	C_18_H_34_O_3_	297.2438	253.21616 (12%)/183.01044 (18%)
	Azelaic acid	C_9_H_16_O_4_	187.0971	169.0856 (2%)/125.09560 (100%)
Amino Acid	L-Tyrosine methyl ester	C_10_H_13_NO_3_	194.0818	179.0577 (8%)/164.0831 (20%)/150.0548 (8%)
Polyene	(9cis)-Retinal	C_20_H_28_O	285.2211	267.2105 (17%)/95.0496 (17%)
Diterpene	(E,E,E)-3,7,11,15-Tetramethylhexadeca-1,3,6,10,14-pentaene	C_20_H_32_	273.2575	163.1480 (79%)/149.1325 (55%)
Triterpene	Betulin	C_30_H_50_O_2_	443.3881	425.3881 (36%)/385.3881 (24%)
	Ursolic acid	C_30_H_48_O_3_	455.3531	409.2443 (74%)/391.2338 (100%)/387.2158 (30%)/319.2263 (30%)
Phenolic	1-(5-Hexyl-2,4-dihydroxyphenyl)ethenone	C_14_H_20_O_3_	254.1748 [M+NH_4_]^+^	237.1636 (11%)/98.9846 (100%)
	1-(2,6-Dihydroxyphenyl)-1,3-dodecanedione	C_18_H_26_O_4_	307.1901	276.1719 (87%)/261.1484 (47%)/233.1533 (43%)/107.0858 (89%)
	p-hydroxy benzoic acid	C_7_H_6_O_3_	137.0244	109.0279 (4%)/93.0332 (33%)
	Gallic acid	C_7_H_6_O_5_	171.0288	153.0154 (92%)/127.0369 (85%)/109.0265 (64%)
	Ellagic acid	C_14_H_6_O_8_	300.9989	257.0100 (36%)/283.9970 (66%)/229.0150 (45%), 185.0241 (40%)
Flavonoids	7-Hydroxy-2-methyl-4H-chromen-4-one	C_10_H_8_O_3_	177.0546	159.0546 (42%)/115.0467 (30%)
	Catechin	C_15_H_14_O_6_	289.0717	245.0814 (12%)/203.0709 (54%)/151.0403 (40%)/125.0238 (31%)/109.0290 (100%)
	Epicatechin	C_15_H_14_O_6_	289.0717	245.0814 (15%)/(203.0709 (65%)/151.0403 (56%)/125.0238 (40%)/109.0290 (100%)
	Quinic acid	C_7_H_12_O_6_	191.0561	191.0565 (7%)/173.0455 (5%)/127.0404 (15%)/111.0459 (8%)/93.0356 (61%)/83.0305 (100%)
	Quercitrin	C_21_H_20_O_11_	447.0933	301.0341 (100%)/151.0027 (8%)
	Quercetin	C_15_H_10_O_7_	301.0354	273.0359 (7%)/151.0031 (100%)/121.0294 (25%)/107.0133 (24%)
	Luteolin	C_15_H_10_O_6_	285.0405	217.0507 (10%)/175.0390 (20%)/151.0033 (15%)/133.0291 (100%)
	Apigenin	C_15_H_10_O_5_	269.0455	151.0032 (20%)/117.0341 (100%)
	Dihydromyricetin	C_15_H_12_O_8_	319.0459	193.0135 (%)/165.0170 (10%)/137.02 (30%)/125.0228 (42%)/109.0296 (30%)

[M+NH_4_]^+^ = adduct from ammonium formate. All of them with (N)CE 30%.

## Data Availability

The original contributions presented in this study are included in the article. Further inquiries can be directed to the corresponding author(s).

## References

[1] Jardim Botânico do Rio de Janeiro https://jabot.jbrj.gov.br/.

[2] Gomes F.T.A., de Araújo Boleti A.P., Leandro L.M., Squinello D., Aranha E.S.P., Vasconcelos M.C., Cos P., Veiga-Junior V.F., Lima E.S. (2017). Biological Activities and Cytotoxicity of *Eperua oleifera* Ducke Oil-Resin. Pharmacogn. Mag..

[3] Leandro L.M., Veiga-Junior V.F. (2012). O Gênero *Eperua* Aublet: Uma Revisão. Sci. Amazon..

[4] Leandro L.M., da Veiga-Junior V.F., Sales A.P.B., do O Pessoa C. (2015). Composição Química e Atividade Citotóxica Dos Óleos Essenciais Das Folhas e Talos de *Eperua duckeana* Cowan. Bol. Latinoam. Caribe Plantas Med. Aromat..

[5] de Sousa J.P.B., Brancalion A.P.S., Junior M.G., Bastos J.K. (2012). A Validated Chromatographic Method for the Determination of Flavonoids in *Copaifera Langsdorffii* by HPLC. Nat. Prod. Commun..

[6] Xavier Junior F.H., Gueutin C., do Vale Morais A.R., do Nascimento Alencar E., do Egito E.S.T., Vauthier C. (2016). HPLC Method for the Dosage of Paclitaxel in Copaiba Oil: Development, Validation, Application to the Determination of the Solubility and Partition Coefficients. Chromatographia.

[7] Souza A.B., Moreira M.R., Borges C.H.G., Simão M.R., Bastos J.K., de Sousa J.P.B., Ambrosio S.R., Veneziani R.C.S. (2013). Development and Validation of a Rapid RP-HPLC Method for Analysis of (−)-copalic Acid in Copaíba Oleoresin. Biomed. Chromatogr..

[8] Bertolucci S.K.V., Pereira A.B.D., Pinto J.E.B.P., de Aquino Ribeiro J.A., de Oliveira A.B., Braga F.C. (2009). Development and Validation of an RP-HPLC Method for Quantification of Cinnamic Acid Derivatives and Kaurane-Type Diterpenes in Mikania Laevigata and Mikania Glomerata. Planta Med..

[9] Al-Sulaiti H., Almaliti J., Naman C.B., Al Thani A.A., Yassine H.M. (2023). Metabolomics Approaches for the Diagnosis, Treatment, and Better Disease Management of Viral Infections. Metabolites.

[10] Cui L., Lu H., Lee Y.H. (2018). Challenges and Emergent Solutions for LC-MS/MS Based Untargeted Metabolomics in Diseases. Mass. Spectrom. Rev..

[11] Couttas T.A., Jieu B., Rohleder C., Leweke F.M. (2022). Current State of Fluid Lipid Biomarkers for Personalized Diagnostics and Therapeutics in Schizophrenia Spectrum Disorders and Related Psychoses: A Narrative Review. Front. Psychiatry.

[12] Lazofsky A., Brinker A., Rivera-Núñez Z., Buckley B. (2023). A Comparison of Four Liquid Chromatography–Mass Spectrometry Platforms for the Analysis of Zeranols in Urine. Anal. Bioanal. Chem..

[13] Li C., Chu S., Tan S., Yin X., Jiang Y., Dai X., Gong X., Fang X., Tian D. (2021). Towards Higher Sensitivity of Mass Spectrometry: A Perspective from the Mass Analyzers. Front. Chem..

[14] Blaženović I., Kind T., Ji J., Fiehn O. (2018). Software Tools and Approaches for Compound Identification of LC-MS/MS Data in Metabolomics. Metabolites.

[15] Ebbels T.M.D., van der Hooft J.J.J., Chatelaine H., Broeckling C., Zamboni N., Hassoun S., Mathé E.A. (2023). Recent Advances in Mass Spectrometry-Based Computational Metabolomics. Curr. Opin. Chem. Biol..

[16] Kontou E.E., Walter A., Alka O., Pfeuffer J., Sachsenberg T., Mohite O.S., Nuhamunada M., Kohlbacher O., Weber T. (2023). UmetaFlow: An Untargeted Metabolomics Workflow for High-Throughput Data Processing and Analysis. J. Cheminform..

[17] Misra B.B. (2021). New Software Tools, Databases, and Resources in Metabolomics: Updates from 2020. Metabolomics.

[18] Souza A.L., Patti G.J. (2021). A Protocol for Untargeted Metabolomic Analysis: From Sample Preparation to Data Processing. Mitochondrial Medicine: Volume 2: Assessing Mitochondria.

[19] Rivera-Pérez A., Garrido Frenich A. (2024). Comparison of Data Processing Strategies Using Commercial vs. Open-Source Software in GC-Orbitrap-HRMS Untargeted Metabolomics Analysis for Food Authentication: Thyme Geographical Differentiation and Marker Identification as a Case Study. Anal. Bioanal. Chem..

[20] Shahzadi I., Nadeem R., Hanif M.A., Mumtaz S., Jilani M.I., Nisar S. (2017). Chemistry and Biosynthesis Pathways of Plant Oleoresins: Important Drug Sources. Int. J. Chem. Biochem. Sci..

[21] Patitucci M.L., Veiga Jr V.F., Pinto A.C., Zoghbi M.G.B., Silva J.R.A. (1995). Utilização de Cromatografia Gasosa de Alta Resolução Na Detecção de Classe de Terpenos Em Extratos Brutos Vegetais. Quim. Nova.

[22] da Silva Antonio A., Oliveira D.S., Dos Santos G.R.C., Pereira H.M.G., Wiedemann L.S.M., da Veiga-Junior V.F. (2021). UHPLC-HRMS/MS on Untargeted Metabolomics: A Case Study with *Copaifera* (Fabaceae). RSC Adv..

[23] Sumner L.W., Amberg A., Barrett D., Beale M.H., Beger R., Daykin C.A., Fan T.W.-M., Fiehn O., Goodacre R., Griffin J.L. (2007). Proposed minimum reporting standards for chemical analysis. Metabolomics.

[24] Cole R.B., Harrata A.K. (1993). Solvent Effect on Analyte Charge State, Signal Intensity, and Stability in Negative Ion Electrospray Mass Spectrometry; Implications for the Mechanism of Negative Ion Formation. J. Am. Soc. Mass. Spectrom..

[25] Arruda C., Aldana Mejía J.A., Ribeiro V.P., Gambeta Borges C.H., Martins C.H.G., Sola Veneziani R.C., Ambrósio S.R., Bastos J.K. (2019). Occurrence, Chemical Composition, Biological Activities and Analytical Methods on *Copaifera* Genus—A Review. Biomed. Pharmacother..

[26] Barbosa K.d.S., Yoshida M., Scudeller V. (2009). V Detection of Adulterated Copaiba (*Copaifera multijuga* Hayne) Oil-Resins by Refractive Index and Thin Layer Chromatography. Rev. Bras. Farmacogn..

[27] do Nascimento M.E., Zoghbi M.G.B., Pinto J.E.B.P., Bertolucci S.K.V. (2012). Chemical Variability of the Volatiles of *Copaifera Langsdorffii* Growing Wild in the Southeastern Part of Brazil. Biochem. Syst. Ecol..

[28] Silva W.G.D., Cortesi N., Fusari P. (2010). Copaiba Oleoresin: Evaluation of the Presence of Polycyclic Aromatic Hydrocarbons (PAHs). Braz. J. Pharm. Sci..

[29] Cavalcanti B.C., Costa-Lotufo L.V., Moraes M.O., Burbano R.R., Silveira E.R., Cunha K.M.A., Rao V.S.N., Moura D.J., Rosa R.M., Henriques J.A.P. (2006). Genotoxicity Evaluation of Kaurenoic Acid, a Bioactive Diterpenoid Present in Copaiba Oil. Food Chem. Toxicol..

[30] Ohsaki A., Yan L.T., Ito S., Edatsugi H., Iwata D., Komoda Y. (1994). The Isolation and in Vivo Potent Antitumor Activity of Clerodane Diterpenoid from the Oleoresin of the Brazilian Medicinal Plant, *Copaifera Langsdorfi* Desfon. Bioorg. Med. Chem. Lett..

[31] Yamamoto T., Yamamoto K. (2005). Accelerator of Collagen Production. US Patent.

[32] Símaro G.V., Lemos M., da Silva J.J.M., Ribeiro V.P., Arruda C., Schneider A.H., de Souza Wanderley C.W., Carneiro L.J., Mariano R.L., Ambrósio S.R. (2021). Antinociceptive and Anti-Inflammatory Activities of *Copaifera Pubiflora* Benth Oleoresin and Its Major Metabolite Ent-Hardwickiic Acid. J. Ethnopharmacol..

[33] Bandara B.M.R., Wimalasiri W.R., Bandara K.A.N.P. (1987). Isolation and Insecticidal Activity of (-)-Hardwickiic Acid from *Croton Aromaticus*. Planta Med..

[34] Royer M., Stien D., Beauchêne J., Herbette G., McLean J.P., Thibaut A., Thibaut B. (2010). Extractives of the Tropical Wood Wallaba (*Eperua Falcata* Aubl.) as Natural Anti-Swelling Agents. Holzforschung.

[35] Braz Filho R., Gottlieb O.R., Pinho S.L.V., Monte F.J.Q., Da Rocha A.I. (1973). Flavonoids from Amazonian Leguminosae. Phytochemistry.

[36] Jiang C., Gates P.J. (2024). Systematic Characterisation of the Fragmentation of Flavonoids Using High-Resolution Accurate Mass Electrospray Tandem Mass Spectrometry. Molecules.

[37] Parailloux M., Godin S., Lobinski R. (2023). Nontargeted Screening for Flavonoids in Salicornia Plant by Reversed-Phase Liquid Chromatography–Electrospray Orbitrap Data-Dependent MS2/MS3. Molecules.

[38] Śliwka-Kaszyńska M., Anusiewicz I., Skurski P. (2022). The Mechanism of a Retro-Diels–Alder Fragmentation of Luteolin: Theoretical Studies Supported by Electrospray Ionization Tandem Mass Spectrometry Results. Molecules.

[39] NIST, Mass Spectrometry Data Center (2017). Tandem Mass Spectral Library.

